# An appeal to magic? The discovery of a non-enzymatic metabolism and its role in the origins of life

**DOI:** 10.1042/BCJ20160866

**Published:** 2018-08-29

**Authors:** Markus Ralser

**Affiliations:** 1 Department of Biochemistry and Cambridge Systems Biology Centre, University of Cambridge, 80 Tennis Court Rd, Cambridge CB2 1GA, U.K.; 2 Molecular Biology of Metabolism Laboratory, The Francis Crick Institute, 1 Midland Rd, London NW1 1AT, U.K.; 3 Department of Biochemistry, Charitè, Am Chariteplatz 1, 10117 Berlin, Germany

**Keywords:** glycolysis, Krebs cycle, metabolism, origin of life, pentose phosphate pathway

## Abstract

Until recently, prebiotic precursors to metabolic pathways were not known. In parallel, chemistry achieved the synthesis of amino acids and nucleotides only in reaction sequences that do not resemble metabolic pathways, and by using condition step changes, incompatible with enzyme evolution. As a consequence, it was frequently assumed that the topological organisation of the metabolic pathway has formed in a Darwinian process. The situation changed with the discovery of a non-enzymatic glycolysis and pentose phosphate pathway. The suite of metabolism-like reactions is promoted by a metal cation, (Fe(II)), abundant in Archean sediment, and requires no condition step changes. Knowledge about metabolism-like reaction topologies has accumulated since, and supports non-enzymatic origins of gluconeogenesis, the *S*-adenosylmethionine pathway, the Krebs cycle, as well as CO_2_ fixation. It now feels that it is only a question of time until essential parts of metabolism can be replicated non-enzymatically. Here, I review the ‘accidents’ that led to the discovery of the non-enzymatic glycolysis, and on the example of a chemical network based on hydrogen cyanide, I provide reasoning why metabolism-like non-enzymatic reaction topologies may have been missed for a long time. Finally, I discuss that, on the basis of non-enzymatic metabolism-like networks, one can elaborate stepwise scenarios for the origin of metabolic pathways, a situation that increasingly renders the origins of metabolism a tangible problem.

## Introduction

To postulate one fortuitously catalyzed reaction, perhaps catalyzed by a metal ion, might be reasonable, but to postulate a suite of them is to appeal to magic. *Orgel [14]*

A century of biochemical research has revealed a stunning conservation of the topological organisation and functional principles of the metabolic network, the biochemical system that enables all life processes [1–5]. This metabolic network provides all the components required for the growth and endurance of cells, and defining their molecular make-up shapes (and defines) the conditions in which life itself can persist and evolve. However, we know very little about the evolutionary origins of the metabolic network [6–8]. This knowledge is missing to explain the basic function principles of metabolism. Furthermore, also solving the origin of life is inevitably bound to create an understanding about how the chemical, functional and organisational principles that define cellular metabolism came into being. Because of the tight intertwining of metabolism and the physiology of the cell, understanding the origins of metabolism is not only important to gain insights into how the first biomolecules formed but also to understand how cells obtained the properties which shape cellular evolution, in which conditions life could persist, and how biological systems function and develop.

In the modern cell, most reactions considered important within the metabolic network — but not all — are protein-enzyme catalysed. The remaining reactions are either spontaneous or non-enzymatic, driven by sunlight, free radical chemistry or metal ions [9]. Driven by the so-called RNA-world hypothesis, there was also extensive research into the possibility of RNA-catalysed metabolic reactions [6,10,11]. Ribozymes that would be important for the core metabolism of any species have so far, however, not been discovered. Additionally, many of the *in vitro* selected ribozymes obtain their catalytic activity via the binding of metal ions such as zinc [12]. If ribozyme-catalysed metabolic reactions exist at all, they account for only a marginal fraction of cellular metabolism.

Protein-based enzymes possess highly sophisticated molecular structures that are a product of Darwinian evolution. A conclusion derived from this situation has been that the metabolic pathway topologies themselves might have emerged through the evolution of enzymes. Following this line of thought, metabolic reaction sequences, as operating in early organisms, would differ substantially from the ones operating in modern organisms [13]. Indeed, until recently, an environmental chemistry that mimics metabolic pathways was not known. The difficulties in identifying such was the basis of claims that such a pathway might not exist, an argument that was used in support of so-called ‘genetics-first’ theories in the origins of metabolism [14–16].

However, the view that metabolic pathways emerge ‘out of the blue’ and solely on the basis of Darwinian evolution creates issues that are difficult to overcome. Darwinian evolution is based on the principle of randomly occurring mutations that are selected if they provide an advantage. The Darwinian process requires a functioning coupling of information encoding and function, hence the presence of a genome or at least an encoding RNA and a translation machinery [17]. These function on the basis of amino acids, nucleotides, ATP or another energy carrier, and NAD(H) or another redox carrier, which are all products of metabolism. Theories that place the origin of metabolism after the origin of the genetic and translation machinery hence create a multi-layered chicken–egg dilemma, for which no sophisticated solution is yet available. A most prominent and early proposed explanation, one still favoured by part of the organic chemistry community active in the origin-of-life field, is that one could form the life-essential molecules initially through another chemistry than the one operating in cells [15,18], a situation that implies a heterotrophic origin of life [19]. A heterotrophic origin, however, creates several evolutionary problems that are difficult to overcome on their own. Often referring to Miller's famous experiment [20], so-called soup theories, for instance, struggle to explain how the origin of metabolism was able to escape equilibrium, a key characteristic of metabolism as a far-from-equilibrium system. Second, it is difficult to provide an answer for how enzymes provided a selective advantage to originate in a Darwinian process. For example, an enzyme cannot provide an advantage in building up an amino acid, for instance, in a soup that already contains the amino acid. Inevitably, an early metabolism would collapse the moment the external supply is exhausted — an anabolic enzyme cannot evolve in the excess of the product it can provide. This left beside — eventually the ‘alternative chemistry’ theory does not solve the problem of its root; essentially, it postpones it: imagining the alternative chemistry is in place, one then still needs to explain the alternative set of chemistry that later becomes metabolism [21]. This brings us right back to the original problem we tried to solve.

Another problem of theories considering a post-genetic origin of metabolism is academically puzzling, and I tend to refer to it as the ‘end product problem’, as stimulated by the first proposed solution to it by Horowitz [22]. Many metabolic pathways involve multiple enzymatic steps to provide the functional products that can be selected in the Darwinian process. If metabolic pathways are to start through a Darwinian process, how was the enzyme that makes the intermediate selected that would be required before the function-providing ‘end’ products can be formed? As it is already unlikely that one complex enzyme comes into being by accident, how are we to explain multiple enzymes coming into being at the same time without providing an advantage before the entire pathway is in place? Horowitz himself suggested the concept, termed ‘retro-evolution’, that could solve the problem. A pathway could just evolve backwards from its last step. Retro-evolution, however, cannot be the entire solution, as it shifts the problem just one step up at a time. In other words, retro-evolution also requires precursors that somehow need to form [23].

There might be a solution for all three problems. What if the precursors to metabolic pathways were not catalysed by RNA or proteins but by the abundant metal ions, and other simple molecules, available on the primordial planet? Well, calling for such networks would be an appeal to magic — would it not?

## Why have bottom-up (chemical) origin-of-life approaches not discovered metabolism-like reactions?

Classically, the ‘origin of metabolism’ problem has been approached with ‘top-down’ approaches and ‘bottom-up’ approaches. In the former, knowledge about metabolic reactions, metabolic pathways, and more recently, metabolic network reconstructions, which have been generated for an increasing number of organisms [24], are used to reconstruct ancestral metabolic pathways or entire networks, down to the Last Universal Common Ancestor (LUCA) [25]. Metabolic network reconstructions have made a huge impact to understand cellular metabolism in biotechnology, and revolutionised metabolic engineering [2,3,26]. They increasingly impact origin-of-life research as well, as they may remove a lot of speculation from the metabolic capacity and habitat of LUCA [25,27,28]. In origin-of-life research, they have, however, one limitation in the sense that they can only provide speculative answers for any situation that preceded LUCA, and that as a difference from biotechnology, one cannot easily test any predictions made [29]. For instance, everyone who ever used genome-scale network reconstruction in the biotechnological context will vividly remember that only a subset of topological predictions prove correct. An often-quoted example is the design of a yeast strain that produces the anti-malaria compound precursor artemisinic acid, which, if rumours hold true, took more than a century of (single-person) trial-and-error time to complete [30].

Despite the huge importance of metabolic reconstructions, one needs to be aware of their nature when interpreting them, specifically in the context of the origins of metabolism. I would like to give some illustrative examples. The first example is the situation that organisms of all kingdoms possess a glycolysis-like oxidative pathway. However, glycolytic enzymes are not sequence-conserved between the kingdoms [31,32]. Hence, sequence-based reconstructions cannot tell whether different glycolytic pathways evolved *de novo* and in parallel after the LUCA or emerged from the same ancestors, with one or both lineages replacing the enzymes. On its own, the reconstruction cannot tell if glycolysis was there before the LUCA or not. Another example is the oxidative and reductive Krebs cycle. Recently, an Archeal citrate synthesis was discovered that operates both in a reductive and oxidative Krebs cycle [33,34]. The metabolic reconstruction can hence not tell whether the early Krebs cycle was reductive or oxidative.

As this restriction seemed difficult to overcome, it led to the ‘bottom-up’ attempts for explaining the origins of metabolism. These attempt to explain the origin of metabolism based on chemistry only, or on a combination of chemistry and biology. Specifically, the former approaches have, however, often struggled to provide tangible insights into the origins of metabolism. Perhaps the best case to illustrate the difficulties of these ‘pure’ bottom-up approaches is: a chemical network that forms multiple metabolites from a hydrogen cyanide precursor [15,35]. Even though some interesting chemistry has certainly been learned in assembling the network [15], it has so far proved difficult to obtain tangible conclusions about the origins of metabolism. For instance, the hydrogen cyanide-based network generated on the basis of organic chemistry is found to be intrinsically incompatible with metabolism, and does hence not provide information about its origin. First, as consecutive reactions cannot occur under the same conditions, the process does not yield a coherent ‘network’ that is to be selected by a Darwinian process. Second, the reactions require conditions so extreme that life could not persist alongside the reactions, preventing the action of evolutionary selection from this angle. Third, the intermediates accumulate to high concentrations by being unable to react further, as they would do in a metabolic process, so that the system quickly enters equilibrium multiple times. Fourth, the topological organisation of the chemical process is found fundamentally different from the one of metabolism, so that cells would need to reprogramme the entire network [15]. Chemical networks, however, intrinsically lack evolvability [36].

Why have the ‘pure’ bottom-up chemical approaches failed to identify reactions that can be attributed to origins of the metabolic network? The hydrogen cyanide chemical network serves as a good case example also to explain this situation. The first reason may be a technical one. A recent benchmark into the analytical procedures used [35,37,38] has revealed that the analytical methods are of too low sensitivity to detect metabolite at their cellular concentration, specifically in the presence of the metabolically important metal ions or oxidants [39]. It is hard to imagine that in the absence of sophisticated enzymes, early forms of metabolism did produce more metabolites as modern forms of the metabolic network. For efficiently screening of protometabolic reactions, the methods would need hence not only reach, but indeed exceed, the sensitivity compared with methods used to study cellular metabolism. In parallel, important geological knowledge was not taken into account considering the choice of analytical techniques. While one does not know much about the prebiotic environment in which early life evolved, the geological sediment record agrees on high Fe(II) concentrations in aquatic environments across the Archean [40–42]. ^1^H-NMR, as implemented in the origin-of-life studies [35,37,38], is however signal-suppressed by the geologically relevant Fe(II) concentrations, specifically in the detection of iron-binding metabolites, such as glycolytic and TCA cycle intermediates [39,43]. Indeed, as discussed below, iron and other metal ions, turned out to be among the key drivers of metabolism like non-enzymatic reactions. Hence, metabolism-like reactions may have been missed as the analytical techniques chosen were not oriented to the biological and geological circumstances, and as a consequence, may have simply not been sensitive enough to detect the a large fraction of the chemical reactions that could have contributed to the origins of metabolism.

Second, the organic-chemistry approach achieves high yields by allowing a reaction to come to completion before the next reaction is induced with a step change in the conditions [15,35]. As attractive as it is from the point of organic chemistry to design reaction topologies in this way, cells during evolution simply do not have the possibility of changing their chemical composition for accommodating different reaction milieus for each of their hundreds of biochemical reactions. Enzymes may, however, evolve very well on the basis of low metabolite levels, so high ‘yields’ may not be required for a chemical reaction to prime enzyme evolution. Hence, the step-change approach itself selects for reactions that are very different from the ones which might be compatible with the Darwinian process; the selection process cannot operate if the reactions cannot co-occur [22,23].

Eventually, the biggest problem may, however, be the lack of applicable constraints, in a principle which I here nickname ‘lost in (chemical) space’. Typically, it is assumed that one has a much higher chance to solve a complicated problem by making use of the available information, rather than excluding it. That is, the more information one has, the easier it is to select from the universe of possibilities, the ones which are relevant for the solution of the problem. Imagine you would like to understand the genius of Mozart. Would it not be counterproductive to exclude history as well as classical music from your research? Or you aim to understand the mysteries of the Cheops pyramid without looking at the architecture of buildings, nor even to ancient Egypt. The chances of solving these problems are much lower in the absence of the key pieces of information. The metabolic network is much more complex than a Mozart piece or the Cheops pyramid however, and its intuitive that knwoledge about how it is structured and how it functions, is helpful in gaining understaning about how it evolved. The particular problem of the unconstrained bottom-up approach is the fact that biological systems use only a very small fraction of the chemical space. Its dimension is best illustrated by pharmacology. It is not unheard off that a drug identification process involves the screening of 50 000 or more chemical compounds to identify a small molecule that eventually functions as a drug. Now, you can create this or even a much larger compound library without any biological knowledge. With a certain statistical probability, the library indeed contains the compound that will eventually cure the disease, i.e. it may be a type of cancer; however, even though one can create the sought-after anti-cancer compound solely on the basis of organic chemistry, without the knowledge of biology, one will not be able to identify the compound among the thousands of ineffective or toxic compounds in the library. Without screening the compounds present in our hypothetical library for binding the biological target or for affecting the phenotype, one would need to conduct the impossible number of 50 000 clinical trials. The most important take-home message from the work of Sutherland et al. may be that origin-of-metabolism research has to face the exactly same problem. One can certainly assemble complex organic-chemical reaction processes that are prebiotically plausible without using any biological knowledge to constrain the search space to those reactions, that are actually compatible with the function principles of metabolic systems. Yet, as the chemical space is much larger than the one used by biological systems, one may very well end up with entire networks of chemical reactions, of which not a single one is compatible with the origins of metabolism.

The final aspect I would like to mention is that a vast majority of chemical literature about the origins of metabolism considers the origins of metabolism as the problem of providing the building blocks for cells. This is not the full picture, however, as the metabolic network determines the chemical and physical properties of living systems, and is integrated in the cellular regulome. Claiming that early life used a fundamentally different chemistry, is hence also a claim that early life would have lived in different conditions, had different intracellular reactions and ionic environment, used different enzymes, used a different chemistry in signalling and nutrient sensing, had different membrane biology, used different transport systems, differed in encoding genetic information, transcription, translation, replication and cell division, and would have responded fundamentally differently to biotic and abiotic stresses.

## The paradox of why extreme thermophiles use metabolic intermediates that are unstable at the temperature they live in — and how the solution to this problem led to the discovery of non-enzymatic iron-dependent glycolysis

My laboratory studies, among other metabolic processes, two central pathways of carbon metabolism: glycolysis and the pentose phosphate pathway (PPP) [44]. We study the function of these core metabolic pathways in microbial fungi (i.e. yeasts) but also in highly similar variants (some are known under different names but overlap largely in topology), including metabolic pathways that convert the glycolytic and PPP set of sugar phosphates that exist across the kingdoms, including in extremophiles [31,45,46]. I was puzzled for a while about the following problem: several of the conserved intermediates, like the essential three-carbon intermediates glyceraldehyde 3-phosphate or dihydroxyacetone phosphate, are highly reactive and degrade at moderate to high temperature within minutes, if not seconds [47,48]. This looked to me to be a huge problem in explaining the metabolism of thermophiles. How can many thermophiles that live at temperatures in which these metabolites are totally unstable use these for some of their most important metabolic reactions? How can an enzyme evolve on the basis of a metabolite that, chemically speaking, should not be there in the given condition? I thought this situation could be very hard to explain both from the evolutionary as well as from the biochemical perspective. However, there is irrefutable proof that these species live and their central carbon metabolism has been shaped and endured millions of years in evolution. Indeed, the discovery of a non-enzymatic glycolysis and PPP provides an answer to this problem that is intuitive. I'll provide the answer below, but first, I need to mention an observation we made.

### Autoclaved yeast growth media is ‘contaminated’ with pyruvate

**Figure 1.**
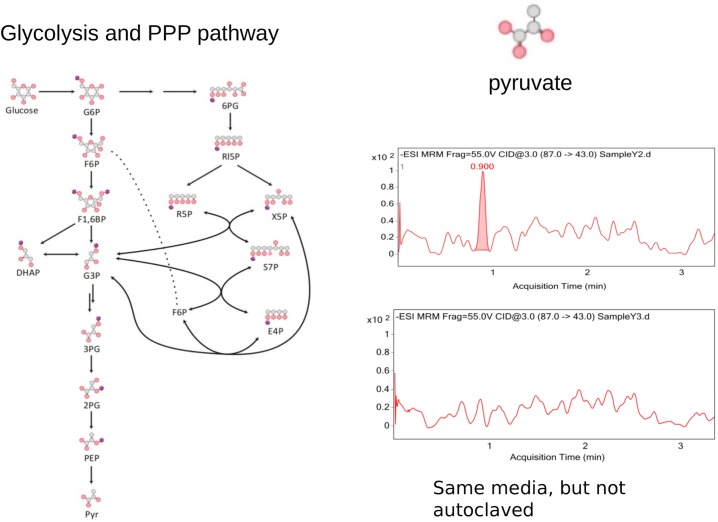
Autoclaved yeast media contains traces of pyruvate, formed non-enzymatically in the heat sterilisation procedure. (Left-hand side) Pathway map of glycolysis and the PPP as occurring in *Saccharomyces cerevisiae*. Modified from [47]. (Right-hand side) Selective reaction monitoring, as conducted in [47], detects pyruvate in yeast synthetic complete media (SC) upon autoclaving it at 121°C for 20 minutes (upper panel).

While we were puzzled about the thermophile problem and just making theoretical assumptions, we made an experimental observation that might (or did) sound unspectacular at first. We discovered that yeast growth media, prepared through a heat sterilisation process known as autoclaving, contains traces of pyruvate (Figure 1). As a metabolism laboratory, we had to trace the source of this contamination, to exclude an influence on our experiments. As the pyruvate signal was not detected in the same solution without the heat sterilisation procedures, we concluded that a heat-induced reaction forms the glycolytic intermediate. As pyruvate is a carbohydrate, potential sources included carbohydrates that are supplied in the yeast growth media such as glucose or citrate. Therefore, something forms pyruvate, potentially from glucose, potentially from citrate, in our yeast growth media. These interconversions (glucose to pyruvate, citrate to pyruvate) did sound familiar to a laboratory working on central metabolism!

We asked the question of whether heat exposure of the yeast media matrix (which is among other things, rich in metal ions) induces reactions that form glycolytic metabolites. As a simple first test, we performed a heat-exposure experiment on the intermediates of glycolysis and the PPP and tested for the formation of glycolytic metabolites by using targeted liquid chromatography-selective reaction monitoring (LC-SRM). LC-SRM is ideal for such investigations. It is a highly sensitive technology with a broad dynamic range in quantification experiments [49]. Important in this context is that LC-SRM can be operated on samples that contain iron (II). Many origin-of-life studies in the past have used other techniques such as ^1^H-NMR implementations that these are signal-suppressed by iron (II) for detecting the metal-binding sugar phosphates and hence cannot detect even highly concentrated glycolytic intermediates [39,43]. However, most of the typical LC-MS/MS studies would also probably have missed glycolysis-like reactions: phosphorylated sugars have poor retention times in many chromatographic methods. As we study glycolytic metabolites frequently, we use an unusual ion pairing reagent, *N*-octylammonium acetate, to achieve a chromatographic separation of the sugar phosphates on reversed-phase chromatography [50,51]. I learned about this specialised method while a postdoc in a Laboratory that was dedicated to the discovery of rare metabolic diseases that are caused by mutations in enzymes of the PPP, and we have progressively worked on improving the method since [43]. I can only speculate, but it is possible that we were not the first ones that tested for non-enzymatic glycolytic reactions in the context of the origins of metabolism, but it is very well possible that we were the first ones that did this using a sufficiently tailored analytical method.

**Figure 2.**
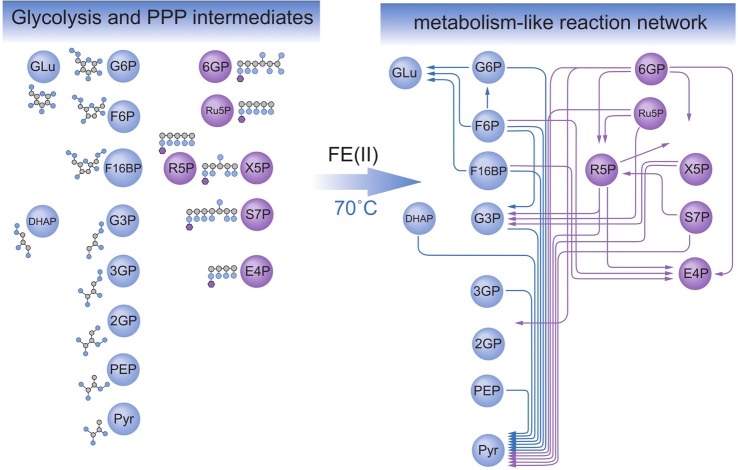
Non-enzymatic glycolysis and PPP-like reactions are enabled by Fe(II), the most abundant transition metal in Archean sediment. Shown is the network that forms at 70°C, while reactions have been detected as starting at 40°C. Modified from [47]. Abbreviations: PPP, pentose phosphate pathway: 6PG, 6-phosphogluconate; Ru5P, ribulose 5-phosphate; R5P, ribose 5-phosphate; X5P, xylulose 5-phosphate; S7P, sedoheptulose 7-phosphate; E4P, erythrose 4-phosphate. Glycolysis: G6P, glucose 6-phosphate; F6P, fructose 6-phosphate; F16BP, fructose 1,6-bisphosphate; DHAP, dihydroxyacetone phosphate; G3P, glyceraldehyde 3-phosphate; 3PG, 3-phosphoglycerate; 2PG, 2-phosphoglycerate; PEP, phosphoenolpyruvate; Pyr, pyruvate.

We observed that most phosphorylated intermediates of glycolysis and the PPP react upon heat exposure to form other metabolites that are found in the pathway, including pyruvate, glucose and ribose 5-phosphate, the RNA backbone sugar. If all reactions are connected, a network graph that resembles the backbone of glycolysis and the PPP is formed (Figure 2).

### How a systematic screen based on Archean sediment constituents identified Fe(II) as a driver of the metabolic reactions

Although the in-water network did already show some similarity with glycolysis, it was not very dense. We were wondering if prebiotic conditions would influence the network, as after all, pure water is a very unlikely matrix for the origins of metabolism. Not knowing much about the primordial planet, we established a collaboration with a geoscience laboratory led by Alexandra V. Turchyn that studies Archean sediment formation at the Department of Earth Sciences, University of Cambridge. We set up a screen to systematically test the impact of the most typical sediment components on glycolytic and PPP intermediates. While testing many salts and metal ions that turned out to have no effect on the reactions, the screen yielded a single hit, and this triggered excitement.

While most of the compounds that we tested had no or very little impact on the glycolysis-like reactions, a very different result was obtained once we added ferrous iron (Fe(II)) to the reaction mixtures. In the presence of Fe(II) in the reaction mixtures (we did work in a low-oxygen atmosphere to keep iron in its reduced and water-soluble form), a much broader spectrum was achieved. More intermediates of glycolysis and the PPP were interconverted, yielding more products that resemble other intermediates of the pathways, and the reactions were faster and more specific (i.e. less carbon was interconverted into other metabolites not part of glycolysis and the PPP). This eventually obtained network covers most parts of glycolysis and the non-oxidative PPP, connecting all implicated intermediate metabolites (Figure 2) [47]. In a follow-up study, we showed that the phosphate group of the glycolytic and PPP intermediates is key for the obtained reactions; it promotes the binding of Fe(II) and the sugar. We also found that pH gradients change the network and allow for the differentiation between glycolysis and the PPP [43].

We speculated that small changes in pH could have enabled early forms of metabolic regulation. Furthermore, another point to mention here is the temperature dependency. While we have shown the initial experiments at 70°C, we also tested lower temperatures. Non-enzymatic glycolytic and PPP reactions already achieve reasonable rates at temperatures of 40°C and lower [47]. Hence, a non-enzymatic metabolism-like reaction sequence that occurs under mundane conditions, as on the basis of a soluble metal cation, is no appeal to magic.

### In non-enzymatic glycolysis and PPP, biology and chemistry match with the geosciences and the principles of Darwinian evolution

In the reducing atmosphere of early Earth, iron stayed in its reduced form and was water soluble. Iron is by far the most concentrated transition metal in Archean sediment. Recent estimates indicate that the Archean oceanic iron concentration may have reached millimolar levels [40,52,53]. There was hence ample Fe(II) available at the time glycolysis or the PPP evolved, and the water-soluble cation would have probably possessed the ability to pass early membrane structures, which presumably were more leaky compared with modern membranes [54]. One might even go one step further and argue that glycolysis and PPP represent the reactions that sugar phosphate intermediates inevitably undergo in an iron-rich solution. Hence, intermediates of glycolysis and the PPP establish a non-enzymatic interconversion network that topologically resembles the pathways of central metabolism. What is equally important however — and that is where the non-enzymatic glycolysis and PPP were distinct from other non-enzymatic processes that were previously associated with the origins of metabolism — is that the network matches the Darwinian requirement; all reactions occur under the same conditions, an essential requirement for the evolution of metabolic pathways that need to form a unit, to provide a selective advantage in any evolutionary process.

### Non-enzymatic metabolism-like reactions may explain why metabolism is functional at temperatures that are beyond the thermal stability of its intermediates

Back to the question of how thermophiles can evolve metabolism that includes metabolites that are unstable at the temperature they live in, such as the small carbon phosphates glyceraldehyde 3-phosphate or dihydroxyacetone phosphate. The solution is non-enzymatic reactions that resemble metabolic pathway topologies: if glyceraldehyde 3-phosphate would decompose randomly, it would indeed be a problem in the evolution to metabolism: first because the metabolite would be ‘lost’ for the cell, and second because no topological organisation of the network would be maintained. The situation is, however, completely different if the heat-induced reaction converts the intermediate into another metabolite that is important for the organism. Under heat, pyruvate forms from glyceraldehyde-3 phosphate [47]. At low temperature, it reacts with its isomer dihydroxyacetone phosphate to form fructose 1,6-bisphosphate [48]. In neither case is the metabolite lost for the cell, nor is the topological organisation of the pathway distorted. Instead, an enzymatic reaction is simply replaced by a non-enzymatic reaction.

Of note, studies based on sequence comparison have often found PPP enzymes lacking in thermophiles and concluded on this basis that they ‘lack’ a PPP [55]. Our data show that one needs to be careful with this conclusion; PPP-like reactions occur non-enzymatically at elevated temperatures. An absence of an enzyme in the thermophile does hence not imply also the absence of PPP-like reactions.

### Catabolism bad and anabolism good? Not if you want to evolve living organisms

In an online search, I came across The Sandwalk blog (sandwalk.blogspot.com/) in which provocative language is used against a good number of high-profile scientific papers, many of them in the origin-of-life and evolution field. If still online, a blog entry named ‘more primordial soup nonsense’ makes nonetheless an interesting read, because it points to a common misconception about the origins of metabolism: While there is an ongoing scientific debate about the many unsolved problems about the origins of catabolic processes [6,8,56], the blogger considers the existence of catabolism trivial (he calls it the ‘wrong’ metabolism), while, in his opinion, only the origin of anabolism has to be considered (he calls it the ‘right’ metabolism). In the view of the blogger, glycolysis has no place in the evolution of metabolism as it converts higher-order carbohydrates into smaller metabolites, but a non-enzymatic gluconeogenesis would be fine, i.e. because it forms carbon bonds.

Irrespective of whether the carbon bond-forming reaction of gluconeogenesis indeed seems to have originated in non-enzymatic chemistry as well [48], the anabolism-only view does not match the structure of the metabolic network. Why, one may ask, if only the anabolic processes would be important, is the metabolic network, irrespective if a species is an autotroph or heterotroph, always a tight intertwining of anabolic and catabolic reactions. Darwinian evolution only selects processes that provide an advantage. If catabolism would have been deleterious at any point in evolution, catabolic pathways would not have evolved. It adds that enzymes are not working wonders, but catalysts. An enzymatic catabolism underlies the same thermodynamic principles as a non-enzymatic catabolism. If the former does not violate thermodynamic principles, so does not the latter.

For understanding the evolution of enzymatic pathways, one has to keep in mind that selective advantage of an anabolic product is only given if a metabolite can be used by the biological system. Certainly, glycolysis could only evolve because biological systems can form glucose, but equally, gluconeogenesis does only provide a selective advantage because the biological system can metabolise the glucose formed.

The other point is that metabolism is a far from equilibrium system. In the context of the origins of life, one likely disequilibrium between CO_2_ and H_2_ has been discussed as a basis of autotrophic carbon fixation [57,58]; but in living cells all energy, redox and ionic concentrations are kept from equilibrium to maintain biological processes. The metabolic network depends on the parallel occurrence of anabolic and catabolic processes to prevent any metabolic system from entering equilibrium. A well-understood example is macro-autophagy, a process in which starved cells metabolize and rebuild their own macromolecules and organelles, to prevent cellular damage, but also, to maintain a constant flow of energy [59]. If early metabolism would be exclusively anabolic, it would enter equilibrium with its environment once all available substrate has been converted. Preventing the system from entering equilibrium is enabled in the metabolic network by being an intertwined system of antagonistic reactions, catabolic and anabolic, oxidative and reductive, endergonic and exergonic. By being bound to the same laws of physics, it is not conceivable that early metabolism was different in this respect: operating far from multiple equilibria is an essence of life. This situation clearly supports the assumption of a synchronous evolution of autotrophy and heterotrophy [60].

The third important point is that thermodynamic properties in a system like in a metabolic network are coupled to one another. A single reaction, or a single pathway, plays its role in life by being part of the metabolic system, which involves both ecological and environmental interactions. What matters are the properties of the system as a whole, that is a sum of the properties of all of its intermediate reactions. It has, for instance, been argued that the Wood–Ljungdahl might be the oldest among carbon fixation pathways because of its exergonic nature [61]. Glycolysis is exergonic too — does it mean it is older than gluconeogenesis, or any process that forms glucose? The question of whether an essential metabolic process is exergonic or requires external energy supply, or whether it is oxidative or reductive, does not answer how old it is evolutionarily. Indeed, globally speaking, metabolism requires constant energy input to keep the network operational far from equilibrium, with its most important source in modern organisms being carbon sources generated photosynthetically. Seen as the intertwined system of coupled reactions that couple across species, the metabolic network as a global system is not, and never was, exergonic.

### A universal principle? Non-enzymatic pendants to gluconeogenesis, the Krebs cycle, glyoxylate shunt, non-enzymatic acetyl-CoA pathway and the SAM pathway

If a non-enzymatic process enabled the evolution of glycolysis and the PPP — but the two pathways alone are not sufficient to form a functional system — this means that additional, perhaps many, non-enzymatic metabolism-like reactions still need to be discovered. Indeed, over the last two years, a plethora of additional metabolism-like chemical networks have been elaborated. I discuss some of these in brief.

#### Non-enzymatic gluconeogenesis

The biological importance of glycolysis requires an evolutionary origin of a process that can also form the intermediate of upper glycolysis. A key candidate is gluconeogenesis, an essential pathway that operates opposite to the catabolic functionality of glycolysis. A recently discovered aldol condensation can form fructose 1,6-bisphosphate from the otherwise unstable glycolytic three-carbon intermediates dihydroxyacetone phosphate and glyceraldehyde-3-phosphate, preventing the metal-induced catabolic reaction that otherwise would form pyruvate [48]. The reaction was discovered in ice, and continues there, over months, to constantly form fructose 1,6-bisphosphate. This does not necessarily mean that gluconeogenesis emerged in ice. The reaction can also proceed through drying–desiccation cycles at higher temperatures. Notable about the non-enzymatic aldol reaction is that it is insensitive to Archean sediment metal ions, but is instead accelerated by simple amino acids such as glycine and lysine [48]. The nature of the non-enzymatic gluconeogenesis-like formation of fructose 1,6-bisphosphate shows that simple condition cycling, i.e. between freezing–thawing (i.e. at Geysers), simple desiccation–dehydration cycles, or cycles between metal ion and amino acid availability, all push a glycolysis/gluconeogenesis system out of equilibrium. There are hence multiple scenarios available, in which glycolysis and gluconeogenesis could have evolved in parallel.

#### Non-enzymatic formation of *S*-adenosylmethionine

Another recently discovered important non-enzymatic reaction leads to the formation of *S*-adenosylmethionine (SAM), the key donor of cellular methyl groups. Enzymatically, the formation of SAM is highly sophisticated, but the Tawfik laboratory recently discovered that ATP and adenosine react non-enzymatically with methionine to yield SAM spontaneously. Again, a non-enzymatic metabolism-like reaction may have primed the emergence of the complex metabolic pathway [62].

#### Non-enzymatic reactions that mimic a reductive Krebs cycle as well as a non-enzymatic acetyl-CoA pathway capable of carbon fixation

Another key metabolic pathway that is canonically referred to as central metabolism is the Krebs cycle. As different from glycolysis and the PPP, the origin of the Krebs cycle has long been discussed in the context of the early evolution of metabolism. In the origin-of-life literature it is typically argued that the early Krebs cycle was most probably reductive, a perception that emerges from the fact that the Early Earth atmosphere was reductive [63,64]. If the Krebs cycle were to run in reverse, which indeed it does in a subset of species [65,66], it provides a route of carbon fixation, which leads to an in part passionate debate about its role in the origins of life [14,67–69]. Indeed, chemical reactions that form Krebs-cycle intermediates have been known for a considerable amount of time. A key debate was initiated by Morowitz et al., who speculated about the prebiotic origin of the reductive Krebs cycle on the basis that multiple Krebs-cycle intermediates are present in the Beilstein database [67,68]. Later, in an elegant organic-chemical study by Zhang and Martin, photoredox chemistry driven by strong UV light and semiconductor particles did enable a series of reductive Krebs cycle-like reactions [70]. In the most comprehensive approach so far, the Moran laboratory presented a suite of 6 out of 11 Krebs cycle reactions that is split in two three-reaction sequences promoted by Zn^2+^, Cr^3+^ and Fe^0^ [71].

More recently, the Moran laboratory also used a similar approach to test for non-enzymatic carbon fixation. This approach was led to the identification of a non-enzymatic Acetyl-CoA pathway [72]. Despite being dependent on native metals, from the topological point of view, the pathway has a strong resemblance to the Wood–Ljungdahl pathway. This seminal work shows that metal-catalysed non-enzymatic carbon fixation in a Krebs cycle-like manner is at least plausible. The approach also beautifully demonstrates the success of bottom-up approaches that instead of dismissing biological knowledge, integrate chemistry with biology.

#### A single-condition network mimicking the Kreb cycle may indicate its origin may have been oxidative after all

The existence of environmental reductive-non-enzymatic Krebs cycle-like reactions is exciting. At the same time, one has to note, however, that despite the intensive three-decade spanning search for reductive Krebs cycle-like non-enzymatic reactions, the ones that have been identified still fall short of providing a precursor for a Darwinian selection process. For the minor part, because these reactions are driven by catalysts such as semiconductor particles, strong UV light, high Cr^3+^ and/or Fe^0^ concentrations if introduced to central metabolism would affect the topological organisation of the metabolic network by catalysing additional reactions that are not part of metabolism. More so, because the reactions require step changes in conditions, a situation arises that forestalls the Darwinian selection of an enzymatic network which essentially requires co-occurrence of the reactions in a single milieu, so that a functional, selectable unit is formed.

Rather than optimizing individual reactions, and aiming to synthesize specific products at optimal yield, we instead screened hence for such unifying reaction conditions. The rationale for the screen was that that if indeed the Krebs cycle emerged from non-enzymatic reactions, a single condition in which many of them can co-occur, must exist. Taken the other way, the approach we choose differs from classic organic-chemistry experimentation [15] in a fundamental point: It takes the biochemical facts (i.e. the identity of the metabolites that participate in the Krebs cycle) as the starting point, and it screens for the component that one is not sure about: the conditions in which the Krebs-cycle intermediates react to form a selectable and coherent network.

It is in the nature of a single-condition approach that, like in metabolism, one expects reactions to occur at different rates, ranging from fast to slow reactions, which requires the application of sensitive analytical techniques with a high dynamic range. The key aspect here is that, for serving as a precursor to the selection process for an enzymatic reaction, a metabolite does not need to be present at high concentrations, but it is important that the reaction can be accelerated by enzymatic evolution to provide an advantage [9].

**Figure 3.**
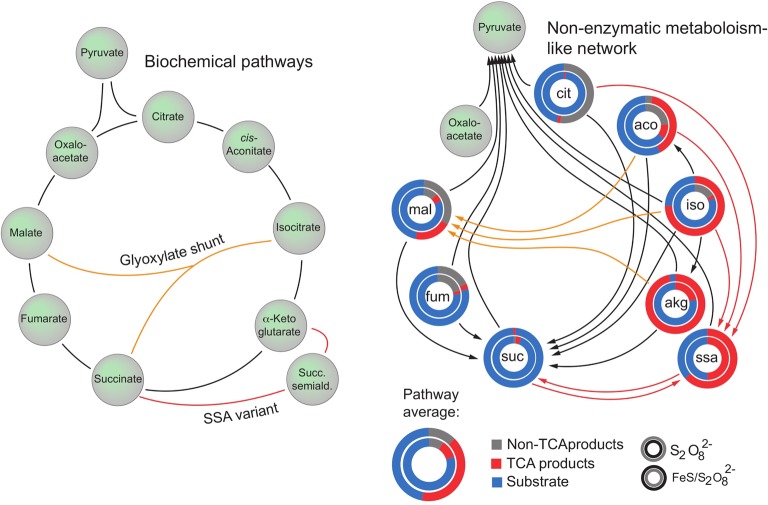
Non-enzymatic Krebs cycle-like reactions that occur in the presence of peroxydisulfate and FeS form a network that closely mimics the typical (oxidative) Krebs cycle and the glyoxylate shunt. (a) Schematic illustration of the enzyme-catalysed Krebs cycle as occurring in yeast (grey), glyoxylate shunt (orange) and succinic semi-aldehyde pathway (red). (b) Non-enzymatic TCA-like reactions replicate large parts of the reaction spectra of the TCA cycle, glyoxylate shunt and succinic semi-aldehyde pathway. Non-enzymatic reactions that occur in the presence of peroxydisulfate and ferrous sulfide are coloured according to whether they replicate the Krebs cycle (black), the glyoxylate shunt (orange) or the succinic semi-aldehyde pathway (red). Circle diagrams illustrate the efficiency in terms of total TCA metabolite recovery of the entire network (substrate formation; blue), TCA-intermediate formation (red) and carbon loss (formation of non-TCA intermediates; grey). The inner and outer circles, respectively, represent peroxydisulfate and the combination of peroxydisulfate and ferrous sulfide. Modified from [74].

We did constrain the screen of a broad range of both reductive and oxidative environments, by the fact that the biology of the Krebs cycle is tightly bound to the presence of iron and sulfur [73]. Literally, we combined a large panel of iron- or sulfur-containing small-molecule compounds with each of the Krebs-cycle intermediates, heated the mixture and used LC-SRM to quantify how many new Krebs-cycle intermediates were formed, eventually in more than 5000 samples. Even though most Krebs-cycle intermediates are stable in water, we could identify a single condition in which all Krebs-cycle intermediates become reactive and interconvert with high specificity into other Krebs-cycle intermediates. The reactions form a Krebs cycle-like non-enzymatic network that, occurring in a single condition, spans over the glyoxylate shunt and the succinic semi-aldehyde pathway ([74], Figure 3). The Krebs cycle has hence non-enzymatic precursors that can be established without step changes in conditions.

The condition identified was broadly seen as a surprise however. While in the case of glycolysis and the PPP the screening approach yielded a condition that met expectations generated by the geosciences (i.e. the driver of the reactions are soluble Fe(II) cations kept reductive in a low- oxygen atmosphere), the unifying condition in which all Krebs-cycle intermediates react in a Krebs cycle-like manner was a solution containing sulfate radicals that are formed from peroxydisulfate — hence an oxidative, and not a reductive condition. In follow-up experiments, we identified also another condition that enables a large fraction of Krebs-cycle non-enzymatic reactions, and also this was oxidative: a solution containing hydrogen peroxide [74]. More recently, a more chemistry-typical study assembled protometabolic analogues of a Krebs cycle. Also here, hydrogen peroxide was found to drive metabolism-like reactions [75].

Initially, were unsure how to interpret this result. While a large fraction in the origin-of-life community currently assumes the Krebs cycle to descend from a reductive pathway, our results as well as the results of Springsteen et al. [75] indicate that oxidising agents have to be considered important players in the origin of the Krebs cycle. Indeed, the oxidative condition is so far the only condition in which every one of the otherwise relatively unreactive Krebs cycle intermediates enters a chemical network that looks much closer to the Krebs cycle compared with anything else observed in the test tube previously (Figure 3). It is important to keep in mind, however, that despite the average equilibrium in the Archean athomsphere was reductive, that H_2_O_2_ and hydroxyl radicals, are essential in metabolism and signalling of every cell [76]. As discussed below, in this context it becomes important to keep in mind that the functional metabolic network is far from equilibrium with its environment. Even in todays oxygenated athmosphere, many metabolic reactions are reductive. Vice versa, all organisms that live in anoxic environments, use oxidative reactions as part of their metabolism.

How strong is actually the evidence that the enzyme-catalysed Krebs cycle originated as a reductive pathway? Phylogenetic data strongly indicate that the Krebs cycle was, at least in parts, already present in LUCA [25]. Phylogenetic data are, however, not conclusive about whether this Krebs cycle was oxidative, reductive or both, as a recently discovered citrate synthase can indicate both directionalities [33,34]. Also, detailed analysis in the ancestral reconstruction of enzyme function has indicated a greater level of substrate promiscuity, and that very few substitutions are required to alter the enzyme's function [77]. Ancestral reconstructions hence need to be seen with great caution when it comes to the extraction of fine specifics, i.e. the reaction kinetics, about an overall conserved pathway.

An often repeated argument centres around the situation that an early atmosphere was reductive [18,67]. In a reductive atmosphere, do only reductive metabolic reactions emerge? This seems not to be the case; several key oxidative pathways date back well into the pre-oxygenation atmosphere, which includes glycolysis and the PPP, which are found in anaerobic as well as aerobic organisms, and much older than the oxygenation of the atmosphere as well [25,78]. So how can oxidative pathways emerge in a reductive atmosphere? This brings us back to the argument that metabolism is a far from equilibrium system, and in a metabolism-like network, reaction properties couple. The origin of an oxidative pathway in a reductive atmosphere is plausible, but it requires a redox disequilibrium, of at least one metabolic reaction. It is worth speculating that modern cells maintain redox disequilibrium through membrane-based compartmentalisation. A hypothetical scenario in the context of early metabolic evolution are hence protocells, which form from simple fatty acids under prebiotically plausible conditions [79,80]. Oxidative reactions are of crucial importance in modern organisms not only to carbohydrate catabolism but also to lipid metabolism, linking them to modern but perhaps also early evolutionary membranes [81,82].

What does the Biological record say about the nature of the early Krebs cycle? First, one notices that many species possess only part of the Krebs cycle. In these cases, the Krebs cycle is typically oxidative, and barely fixes CO_2_ [83,84]. In general, it is not the CO_2_ fixation function that is the common function of the Krebs cycle. Indeed, rather than gaining carbon from the Krebs cycle, the typical problem higher organisms have with the Krebs cycle is actually the loss of CO_2_ induced by its oxidative activity. They use complex mechanisms, including the Cori cycle, rather than a reductive Krebs cycle, to overcome extensive carbon loss. The evolution of complex and costly compensatory mechanisms for the CO_2_ loss as induced by the Krebs cycle seem to be in contrast with the interpretation that its evolutionary precursor was actually providing selective advantage exactly through providing CO_2_ to these species.

Particular attention should be placed on species that possess the Krebs cycle in parallel to other pathways of CO_2_ fixation. Particularly illustrative are photosynthetic species that, despite possessing a fully functional Krebs cycle, use the much slower Calvin cycle for CO_2_ fixation. Keeping in mind that Darwinian evolution can only select for an enzymatic pathway if it provides a selective advantage above the existing metabolic system, this fact is indicative about one of two scenarios. Scenario one is that the Krebs cycle - at least in these species - was not CO_2_ fixating, at least at the time when the Calvin cycle evolved. Scenario two is that photosynthetic organisms lost the ability of fixating CO_2_ through the reductive Krebs cycle at some point, paving the way of the slower Calvin cycle to evolve later. The second scenario would be rather unusual; evolution typically does not take steps back to lose an efficient pathway to replace it with a less efficient one. There remains the possibility that an enzyme-catalysed reductive Krebs cycle could not be maintained mechanistically upon the oxygenation of the atmosphere. But here are photosynthetic species that use a reductive Krebs cycle, indicating the pathway is not necessarily dysfunctional in photosynthetic species [66]. Third, the scenario of ‘replacing’ an early reductive Krebs cycle with the Calvin cycle dictates that, prior to the oxygenation of the atmosphere, the early Krebs cycle would have had no other function than fixing CO_2_ (the Calvin cycle can only replace this function — the two pathways do not overlap in any other intermediates); the essential function of the Krebs cycle in modern organisms is, however, explained by its role of providing intermediates, mainly for the synthesis of amino acids — a process in which the Calvin cycle is not involved. In parallel, a difficulty is that it would dictate the defining reaction of the Calvin cycle, catalysed by Ribulose-1,5-bisphosphate carboxylase (Rubisco), to emerge only upon the oxygenation of the atmosphere. The enzyme can, however, accept not only CO_2_ but also oxygen as a substrate, and even the highly sophisticated modern Rubisco enzyme can only partially prevent this side reaction, explaining why despite immense evolutionary pressure causing Rubisco to have become the most abundant enzyme in the biosphere, the reaction remains so slow in the oxygen-rich atmosphere [85–87].

In other words, the biological evidence about the nature of the early versions of the Krebs cycle sends mixed messages. In particular, it is important to keep in mind that the fact that an early atmosphere was in a reductive equilibrium is no convincing argument towards a claim that all early metabolic reactions were reductive too. This situation underlined by the existence of multiple oxidative pathways like glycolysis, which originated pre-oxygenation by participating in the far from equilibrium metabolic network. More than anything, the debate about the nature of the early Krebs cycle shows that the theoretical fundament about the origins of metabolism, and the conditions that led to the origins of metabolic pathways, are currently far too weak to dismiss systematically obtained experimental evidence, specifically if it is in conflict with some of current hypothetical assumptions.

### Non-enzymatic chemical networks enable a stepwise scenario for the origins of metabolic enzymes

Last but not least, I would like to briefly speculate about the role of non-enzymatic metabolism-like reactions on the origin of the metabolic network. Basically, there are two questions to answer, and these may not be the same. The first one is, were these the processes that also supplied the early cells with their required metabolites? And second, did such reactions enable the evolutionary origins of enzymes, metabolic pathways and, hence, the metabolic network? Obviously, there is no answer to the first question (yet), and indeed, the proof of this principle is still missing. The problem here is that non-enzymatic glycolysis, PPP and oxidative Krebs cycle non-enzymatic reactions [43,47,74,88] can indeed occur in a unifying and mundane condition as required by Darwinian evolution. Each of the three pathways do not, however, at least in their current status, represent a functional metabolic system. In contrast, the non-enzymatic reductive Krebs cycle and the non-enzymatic acetyl-CoA pathways [71,72] have properties of a functional metabolic system; however, they depend on non-metabolic catalysts like native metals and require step changes in conditions, situations that would forestall Darwinian evolutionary processes. In essence, there is rapidly accumulating evidence that metabolism-like non-enzymatic reactions might have been able to supply early cells with their metabolic capacity, but clearly there remains work to be done before one has irrefutable proof.

One might be much closer to an answer to the second question. A key answer the bottom-up approaches must provide is how the chemical network serves in the evolution of enzyme-catalysed metabolism. Non-enzymatic reaction systems may be an elegant solution to overcome the end-product problem: the existence of non-enzymatic precursors converts the origin of a metabolic pathway into a stepwise problem [23], which may be an alternative to the retro-evolution concept for overcoming the aforementioned end-product problem [22]. In the presence of a non-enzymatic network, enzymes can provide a selective advantage by coming into being one step at a time, that is, if they improve the network, starting from its most limiting step. Importantly, the non-enzymatic aldol reaction *in ice* [48] has shown that an amino acid as simple as glycine can fulfil this role in the formation of important metabolites as fructose 1,6-bisphosphate. Amino acids have further been shown to serve as asymmetric catalysts, which may contribute towards solving the long-standing question about what broke the symmetry between d-sugars and l-amino acids [89], or provide evidence about early oxygen fixation reactions [90]. The first ‘enzymes’ may thus have been as simple as single amino acids, complementary, or in complex, with metal ions.

## Concluding remarks

Until recently, non-enzymatic, prebiotic reaction sequences that resemble metabolism were not known. The recent discovery of a non-enzymatic glycolysis and PPP, followed by the discovery of several other non-enzymatic and prebiotically plausible metabolism-like processes, has changed this situation. Hence, it appears that the topology of metabolism is rooted in the non-enzymatic chemistry accessible to early life forms that existed irrespective of (and hence, before) the Darwinian selection process. For glycolysis and the PPP, the key enabling agents are Fe(II) cations, broadly available on the primordial planet, as evidenced from the sedimentary record. Hence, it appears that mundane conditions were implicated in shaping the topological organisation of metabolism, and yet, the existence of non-enzymatic metabolism-like reactions may explain why modern thermophiles can persist at temperatures that are beyond the thermal stability of some of their key metabolites. By serving as a ‘template’ in the Darwinian selection processes, the existence of non-enzymatic metabolism-like reaction sequences may have allowed life to overcome the end-product problem and facilitated the origin of metabolic pathways. Furthermore, there is growing evidence that enzymes might have exploited amino acid-based as well as metal ion catalysis since the beginnings of metabolism. Hence, the origin of life, often dismissed as a ‘philosophical problem’, is increasingly becoming a problem that is experimentally tangible.

## Abbreviations

LC-SRM: liquid chromatography-selective reaction monitoring
LUCA: Last Universal Common Ancestor
PPP: pentose phosphate pathway
SAM: *S*-adenosylmethionine
